# Assessing the generation and dispersal of respiratory particles using upper respiratory commensal bacteria as index organisms for respiratory pathogens

**DOI:** 10.1038/s41598-025-28373-z

**Published:** 2025-12-24

**Authors:** Patricia Barkoci, Wilhemina D’Costa, Neville Q. Verlander, Nicola Yaxley, Ginny Moore

**Affiliations:** 1 Biosafety, Air and Water Microbiology Group, UK Health Security Agency, Porton Down, Salisbury, SP4 0JG UK; 2 Statistics, Modelling and Economics Department, UK Health Security Agency, London, NW9 5EQ UK

**Keywords:** Applied microbiology, Public health

## Abstract

There is a need to better understand the dispersal and spread of respiratory pathogens. However, carrying out controlled microbiology-based simulation studies is difficult, particularly if the target organism is highly pathogenic or is currently unknown. An alternative approach is to use commensal upper respiratory tract (URT) bacteria as index organisms. Healthy volunteers were asked to perform defined respiratory activities within a purpose-built flexible-film isolator. The dispersal of large (L-RPs) and small respiratory particles (S-RPs) was assessed via passive (settle plates) and active air sampling, respectively. Activities were carried out with or without a face covering. Dispersal of respiratory particles differed with individual. During speech, increased vocal volume was associated with a significant increase in the number of URT bacteria deposited as L-RPs and captured by air samplers (S-RPs). Disposable (IIR) medical face masks significantly reduced deposition of L-RPs and reduced the odds of recovering higher numbers of S-RPs by 87% (CI: 63%, 96%). Transparent face coverings were as effective as (IIR) face masks, but face shields had limited efficacy, particularly when worn by individuals who dispersed high numbers of RPs. Commensal URT bacteria can be used to study the risk of respiratory transmission in the absence of the target pathogen.

**Supplementary Information:**

The online version contains supplementary material available at 10.1038/s41598-025-28373-z.

## Introduction

The urgency of the COVID-19 pandemic highlighted knowledge gaps among the scientific community regarding the transmission of respiratory pathogens. This, in turn, catalysed a surge of research activity and a substantial expansion of scientific literature. During the early phases of the pandemic, approximately 10,000 COVID-19-related articles were submitted to scientific journals each month^1^. However, pivotal questions regarding the mechanisms of respiratory particle generation and dispersal, including the potential for ‘super-spreaders’ and for ‘super-spreading’ events (i.e. respiratory activities that could facilitate transmission) continue to warrant investigation.

Dispersal of respiratory pathogens can occur when particles containing potentially infectious organisms are expelled from the upper and lower respiratory tracts of infected individuals. These ‘infectious respiratory particles’ (IRPs) can travel through the air and infect individuals via inhalation (Supplementary Figs. 1a and 1b) or by directly depositing onto facial mucosal membranes (Supplementary Fig. 1c). The deposition of IRPs on hands and/or surfaces could also lead to direct (Supplementary Fig. 1 d) or indirect (Supplementary Fig. 1e) fomite transmission^2^.

The risk of IRP transmission depends on a complex interplay between environmental conditions (e.g., temperature, relative humidity, airflow), the physicochemical characteristics of the pathogen, social behaviour, and mitigation strategy. The activity by which IRPs are expelled by the individual is also important. Expiratory activity has been shown to affect the size^3^ and the concentration of IRPs and the distance they travel^4^.

Previous studies have monitored the number, mass, and size distribution of the particles exhaled by (usually) healthy study participants to risk assess different activities, including singing^5–8^, the playing of musical instruments^9–11^, loud talking^4,12^, and sneezing^13,14^. The effectiveness of face coverings in mitigating the transmission of IRPs has also been assessed across a range of expiratory activities^15–18^.

Non-microbiological techniques, such as those used in the aforementioned studies, allow different activities and mitigation strategies to be directly compared. However, studies must be carried out within an environment with a very low (near-zero) background particle concentration^19^ and sampling must be carried out as close to the source as possible, providing limited information about potential dispersal and spread. They also provide limited information about potential infectivity – if, for example, the particles detected carry (or could carry) the target organism.

Pathogen-based studies using naturally infected participants have demonstrated the dispersal of respiratory pathogens during expiratory activities such as coughing^20^, talking^21^ or breathing^22,23^, and have confirmed the utility of face coverings in mitigating this spread. Whilst these studies provide crucial validation for the role of expiratory activities and source control measures, they are technically and ethically challenging.

An alternative approach is to utilise index organisms. For the purposes of this study, we define *index organisms* as microorganisms originating from the same anatomical source as a given pathogen, such that their detection within a specific environment indicates the potential presence or risk of that pathogen.

To assess the risk of IRP transmission in situ without involving infected individuals, previous studies have utilized oral streptococci as key index organisms. Duguid^24^ and Hamburger and Robertson^25^ investigated the dispersal of oral streptococci during respiratory activities such as talking, coughing, and sneezing. Bennett et al.^26^ utilised airborne streptococcal counts to evaluate the occupational risk associated with dental procedures, whilst Reid et al.^27^ and Torrey and Lake^28^ established a correlation between the concentration of oral streptococci in the air and the incidence of measles and the common cold, respectively.

The oral cavity and upper- and lower respiratory tracts share highly similar microbial communities in healthy individuals^29^, and studies utilising molecular^30^ and culture-based assays^31,32^ have shown that respiratory pathogens such as SARS-CoV-2 are frequently present in saliva and/or the upper respiratory tract (URT). We hypothesize that URT bacteria, which are readily detected in oral fluids, could serve as effective proxies (index organisms) for studying the dispersal of respiratory pathogens. Such an approach would mitigate some of the ethical and technical challenges posed by direct pathogen detection and allow the risk of respiratory transmission to be studied in the absence of a target pathogen.

To demonstrate the utility of this method, we designed a bespoke flexible-film isolator to examine the generation and dispersal of URT bacteria in relation to individual, respiratory activity and mitigation strategy, specifically the effectiveness of face coverings. Results were validated against methodologically more complex studies published in the literature.

## Materials and methods

### Isolator for measuring aerosol and droplet generationn (IMADGENN)

**Fig. 1:**
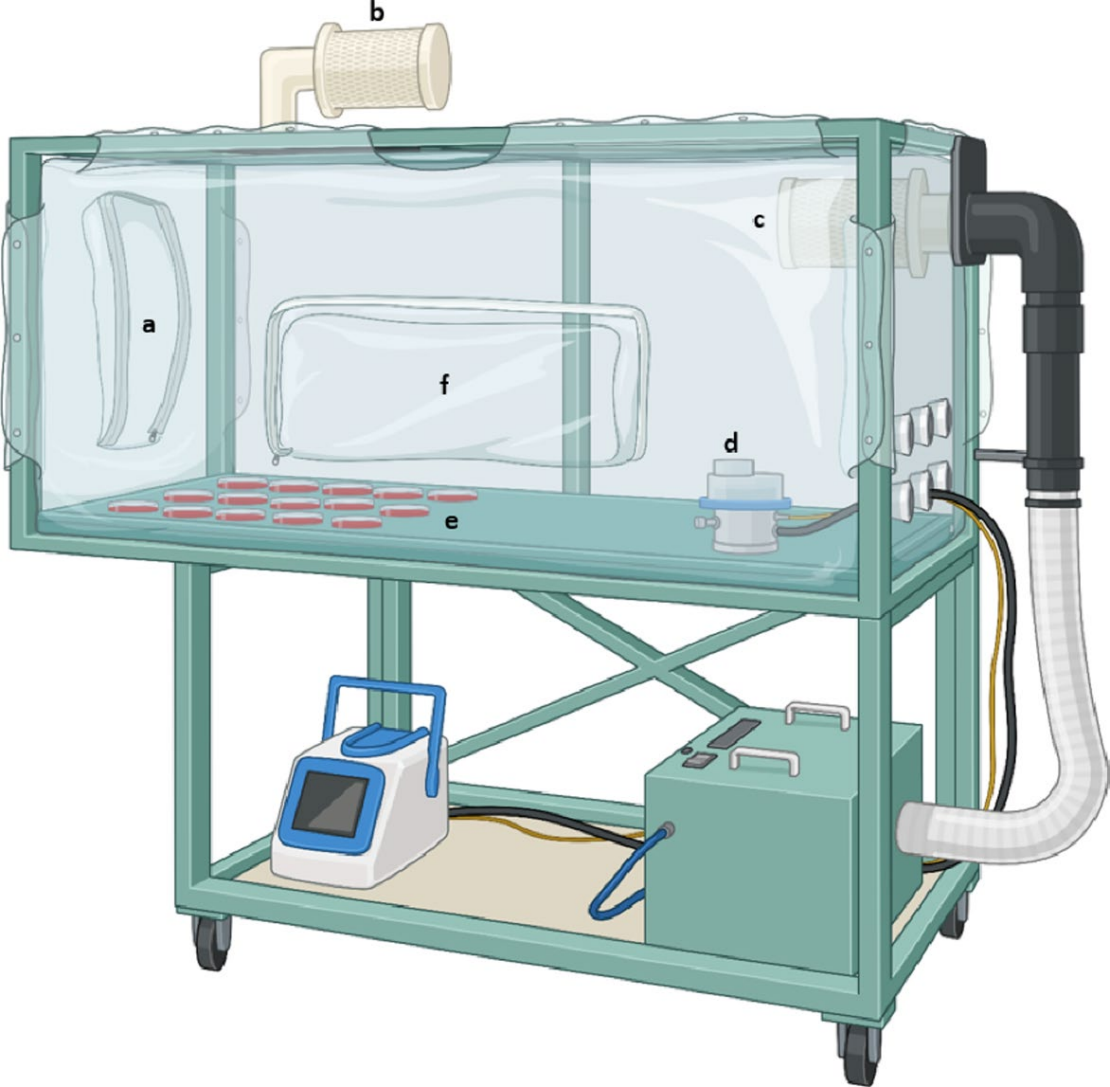
Isolator for Measuring Aerosol and Droplet GENerationN (IMADGENN): front participant panel (**a**), supply HEPA filter (**b**), extract HEPA filter (**c**), air sampler (**d**), settle plates (**e**), side panel (**f**). Created in BioRender.com.

IMADGENN is a purpose-built flexible film isolator (PFI Systems Ltd, Milton Keynes, UK) designed to capture respiratory particles generated by study participants (Fig. 1). The isolator measures 0.5 m (w) x 1.5 m (l) x 0.6 m (h) and is supplied with HEPA-filtered air. The isolator is fastened to a stand (increasing the overall height of IMADGENN to 1.7 m), and non-gas-tight zips allow panels located at the front and the side of the isolator to be opened, facilitating both study participation and the manipulation of sampling equipment. A second HEPA filter (with an efficiency of 99.995% at 0.3 μm) attached to the extract system allows rapid removal of airborne particles and ensures a low level of background aerosol. With the extraction fan operating, the air flow through IMADGENN is 1.72 m^3^/min (280 air changes/hour).

### Participants and experimental setup

This study was approved by the UK Health Security Agency (Public Health England at the time) Research Ethics and Governance Group (reference number R&D 406). All experiments were performed in accordance with relevant guidelines and regulations. Informed consent was obtained from all volunteers prior to participating in the study, when they were also asked to provide their age. No other personally identifiable information was collected, and none was stored.

This study was designed as a proof-of-concept, exploratory investigation to demonstrate the feasibility of using upper respiratory tract (URT) bacteria as proxies for respiratory pathogen dispersal. As such, no formal a priori sample-size calculation was performed. Cohort sizes were determined based on the availability of volunteers and operational constraints (Cohort 1, *n* = 16; Cohort 2, *n* = 15). The study is primarily hypothesis-generating and intended to inform the design of future, fully powered investigations.

Prior to participating in the study, healthy volunteers were asked to self-test for COVID-19 using a lateral flow device and report a negative test result. All study participants provided a saliva sample immediately before completing the experiment to confirm the presence and concentration of URT bacteria.

Before commencing each experimental run, the air filtration system of IMADGENN was operated for three minutes to remove any residual particles. Columbia Blood agar plates (CBA; E & O Laboratories Ltd, Bonnybridge, UK) were positioned inside the isolator at 10 cm intervals (up to 1 m) to the left, centre and right of the isolator (Fig. 1). An active air sampler, an R2S slit-to-agar sampler (Emtek LLC, Longmont, US) operating at a flow rate of 17–28.3 L/min and collecting onto CBA, was positioned inside the isolator 1.2 m from the participant. The lids of the settle plates were removed, and the isolator was secured by zipping up both the side and front panels. The air filtration system continued to operate for at least three minutes to remove particles introduced by the investigator. It was then switched off.

Each participant was positioned directly in front of IMADGENN and was instructed to adjust the chair so that their face was centred adjacent to the front panel (Fig. 1(a)) and approximately 30 cm from the top of the isolator. The air sampler was operated remotely, and the participant was directed to open the panel and to perform the specified activity.

Each experimental run included a fallow period when, following completion of the respiratory activity and with the front panel closed, the air sampler continued to operate. This was to ensure that particles remaining in the air were captured. The air filtration system of the isolator was then switched on and allowed to run for five minutes before the side panel was opened and the agar plates collected and replaced.

### Respiratory activities

For the purposes of this study, URT bacteria that deposited on surfaces (settle plates) were referred to as large respiratory particles (L-RPs), while URT bacteria that remained suspended in the air and captured by the air sampler were termed small RPs (S-RPs).

Here, the term ‘dispersal’ refers specifically to the release or shedding of L-RPs and S-RPs from participants during respiratory activities. It does not refer to the subsequent transport or movement of these particles in the environment.

### Assessing the impact of increased vocal effort (loudness; cohort 1)

Volunteers aged between 21 and 60 years (*n* = 16; 8 female) were asked to count from one to 100 in their ‘normal’ conversational voice. They were then asked to repeat the activity whilst shouting as loud as was comfortable at a sustained amplitude. No face covering was worn. A decibel (dB) meter (Cirrus Research Plc, Hunmanby, UK) was placed inside the isolator (0.8 m from the participant) and used to monitor sound level. The mean, minimum, and maximum dB level was recorded. Each experimental run, including the fallow period, was four minutes.

### Assessing the effectiveness of face coverings in reducing dispersal (cohort 2)

Volunteers aged between 20 and 65 years (*n* = 15; 7 female) were asked to perform a series of respiratory activities, specifically: to cough three times, count from one to 100, exhale through the mouth, sing “Happy Birthday”, exhale through the nose, count from one to 100, and cough three times again. Each participant carried out the same series of respiratory activities when wearing and when not wearing a face covering. Each experimental run, including the fallow period, was 15 min.

**Fig. 2:**
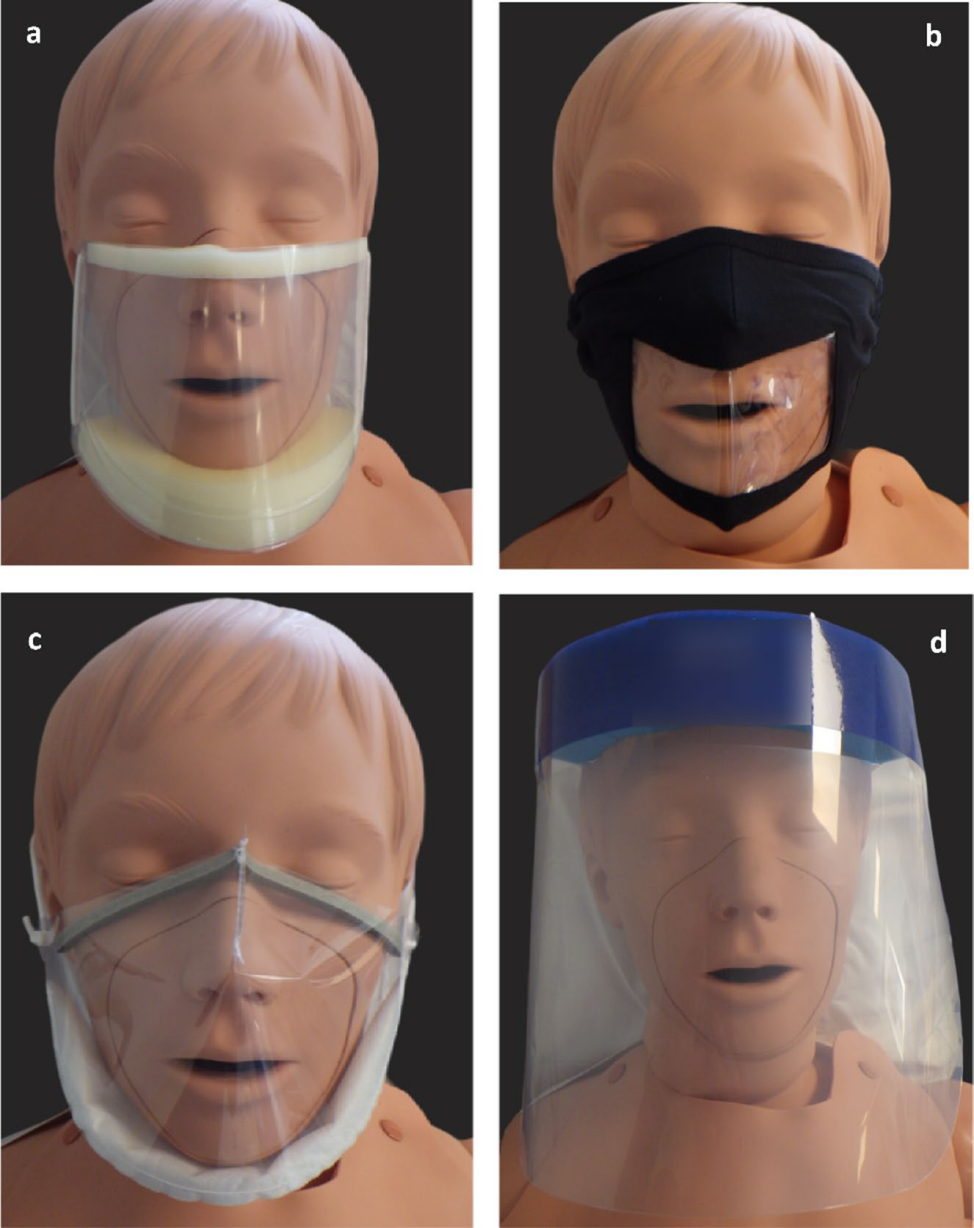
Transparent face coverings: (**a**) ClearMask™ (ClearMask LLC, Baltimore, USA), (**b**) reusable clear window mask (Stevenage Packing Limited, Hertfordshire, UK), (**c**) Panoramic Mio-Mask (L.J.A MIERS & Co. Ltd, Cambridgeshire, UK), (**d**) face shield (SciQuip Ltd, Shropshire, UK).

Three transparent face coverings were selected based on style and commercial availability: the ClearMask™ (ClearMask LLC, Baltimore, USA), a reusable clear window mask (Stevenage Packing Limited, Hertfordshire, UK), and the Panoramic Mio-Mask (L.J.A MIERS & Co. Ltd, Cambridgeshire, UK; Figure 2). The effectiveness of these face coverings in reducing the dispersal of large- and small RPs was compared to that of a disposable (Type IIR) medical face mask (Wuxi Yushou Medical Appliances Co., Ltd, Jiangsu, China) and a face shield (SciQuip Ltd, Shropshire, UK; Figure 2d). Each participant was provided with a disposable (IIR) medical face mask and two of the other four face coverings, the selection of which was assigned at random. The sequence in which the face coverings were worn was also determined by random allocation. However, in all instances, the participant first carried out the series of respiratory activities whilst not wearing any face covering.

### Culture and identification of URT bacteria

Each saliva sample was serially diluted (10-fold) in sterile water, and aliquots (100 µl) of the 10^− 5^ and 10^− 6^ dilutions were cultured in duplicate on CBA. All saliva samples were then disposed of, and all CBA plates, including those removed from IMADGENN, were incubated at 37 °C for 48 h.

After incubation, bacterial colonies were counted and presumptive URT bacteria identified by matrix-assisted laser desorption/ionization time-of-flight (MALDI-TOF) mass spectrometry (Bruker Daltonik MALDI biotyper; Bruker, Bremen, Germany) using the extended direct transfer method.

### Data analysis

Descriptive and non-parametric analysis was performed using GraphPad Prism 9 or R (version 4.3.3). Further data manipulation and inferential analysis were performed in STATA, version 17.0.

As each participant contributed multiple measurements, mixed effects models were used, with participant as the random effect and the remaining variables as the fixed effects. Following the conclusion of the modelling process, if the random effect was practically indistinguishable from zero, it was removed, after which the measures of effect (odds ratio (OR) or relative risk ratio (RRR), as appropriate), together with their 95% confidence intervals (CIs), were obtained. Where possible, the likelihood ratio test was used to obtain p-values; otherwise, the p-values were obtained by means of the Wald test. Statistical significance level was taken to be 5%.

Ordinal logistic regression was used, fitting a generalised ordinal logistic regression model at the end of the modelling process, when the test for proportionality of odds as implemented by Williams^33^ or, where not possible, the approximate likelihood ratio test, as described by^34^ Wolfe and Gould indicated a possible violation of the assumption.

The amount of data available determined the analysis strategy. This and the modelling procedure are described in full in the supplementary material.

## Results

### Index organisms for respiratory pathogens: commensal URT bacteria in saliva and respiratory particles

The mean concentration of URT bacteria recovered from the saliva of the participants ranged from 3 × 10^6^ to 2.9 × 10^9^ CFU/ml (cohort 1) and from 1.5 × 10^7^ to 2.5 × 10^8^ CFU/ml (cohort 2). *Neisseria* spp. (comprising species *flavescen*,* perflava*,* subflava*,* mucosa*,* elongata*,* macacae*) and *Streptococcus* spp. (*cristatus*,* sanguinis*,* parasanguinis*,* mitis*,* salivarius*,* oralis*,* pneumoniae*,* australis*,* infantis*,* vestibularis*,* gordonii*) were the most common URT bacteria present both in saliva and in the respiratory particles produced by all participants. Other predominant URT commensals were *Rothia *spp (*aeria*,* mucilaginosa*,* dentocariosa*), *Actinomyces *spp (*naeslundii*,* oris*,* odontolyticus*), *Gemella * spp(*haemolysans*,* sanguinis*), *Haemophilus parainfluenzae*, and *Moraxella *spp.

### The impact of increased vocal effort (loudness) on the dispersal of RPs

#### Large respiratory particles (L-RPs)

**Fig. 3:**
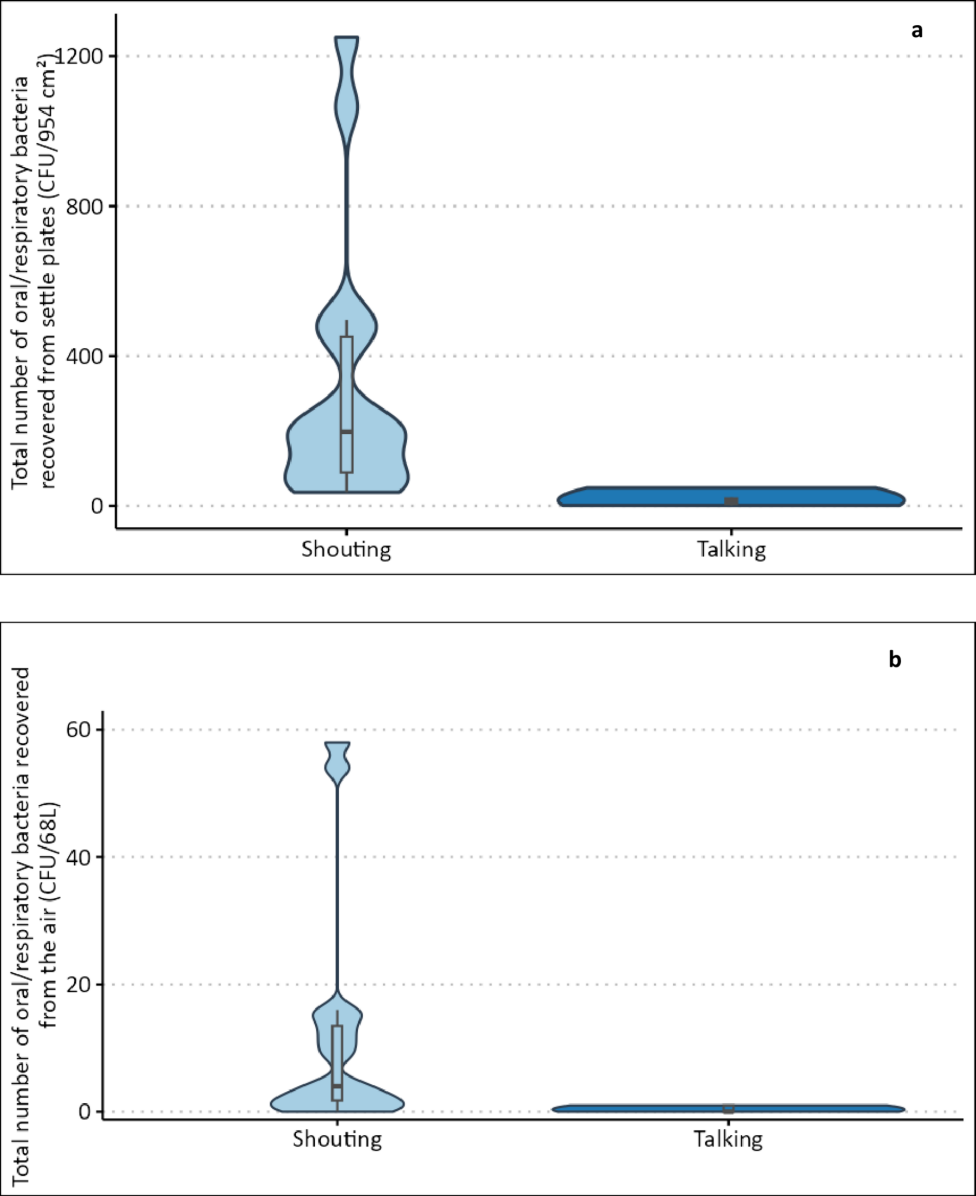
Impact of increased vocal effort (loudness) on the dispersal of small and large respiratory particles (S-RPs and L-RPs). Healthy volunteers (*n* = 16) were asked to count from 1 to 100 when shouting or in their normal conversational voice. Truncated violin plots with box plot illustrate the total number of URT bacteria deposited on surfaces within 0.5 m from the source (L-RPs) (**a**) and captured by the air sampler (S-RPs) positioned 1.2 m from the source and operating at 17 L/min (**b**).

**Fig. 4:**
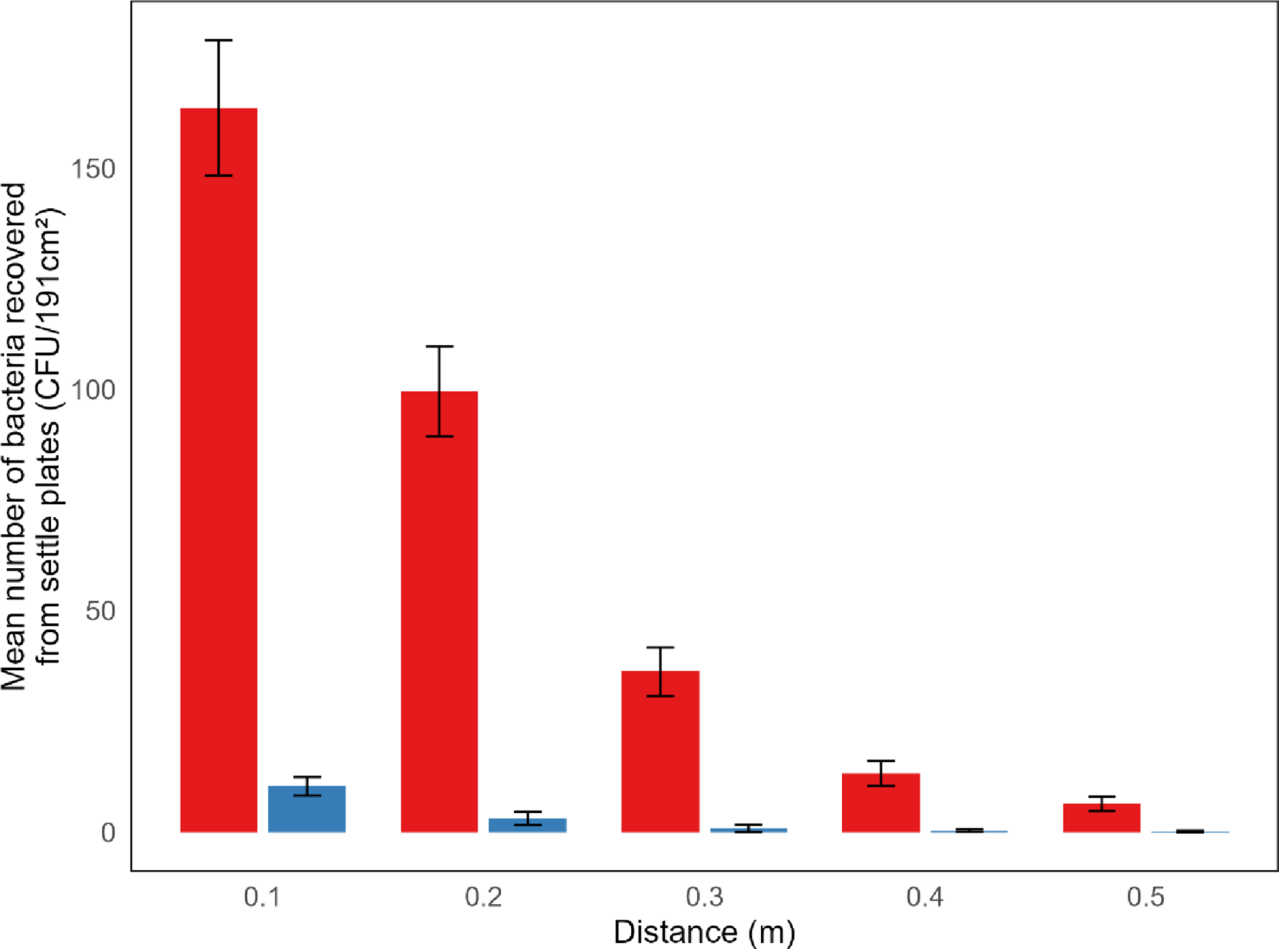
The mean number (± SD) of small and large respiratory particles recovered from droplets deposited on surfaces at increasing distance from the source. Healthy volunteers (*n* = 16) were asked to count from 1 to 100 when shouting (red bars) or in their normal conversational voice (blue bars).

When volunteers (*n* = 16) were instructed to count from 1 to 100 in their normal conversational voice, the mean dB level measured within IMADGENN was 75 dB, and the number of URT bacteria deposited on settle plates within 0.5 m from the source ranged from 1 to 48 colony-forming units (CFU) with a median of 13 CFU (Fig. 3a). When the volunteers were instructed to repeat the counting while ‘shouting’, the mean dB level recorded was 92 dB, equating to an increase of 15 dB and 17 dB for males and females, respectively. This increased vocal effort resulted in a 15-fold increase in the median number of URT bacteria deposited as L-RPs (range: 36 CFU − 1251 CFU (median 197 CFU); Fig. 3a). Deposition significantly decreased with increasing distance from the source (*p* < 0.001; Fig. 4).

#### Small respiratory particles (S-RPs)

Without considering other factors (univariate analysis), increased vocal effort (‘shouting’) was associated with a significant increase in the number of URT bacteria captured by the air sampler (S-RPs; *p* = 0.01) and a significant linear association between mean dB level and the concentration of URT bacteria recovered as S-RPs. Some individuals dispersed comparatively more S-RPs (Fig. 3b). However, after adjusting for age, activity, and height, the odds of recovering a higher number of URT bacteria as S-RPs increased by 34% for every dB increase in mean voice level (95% CI: 1.06, 1.70; *p* = 0.003).

#### The effectiveness of face coverings in reducing the dispersal of RPs

When volunteers (*n* = 15) were asked to perform a series of respiratory activities without a face covering, the median number of URT bacteria recovered as L-RPs and deposited within 1 m from the source was 84 CFU (range: 6 CFU – 978 CFU).

**Fig. 5:**
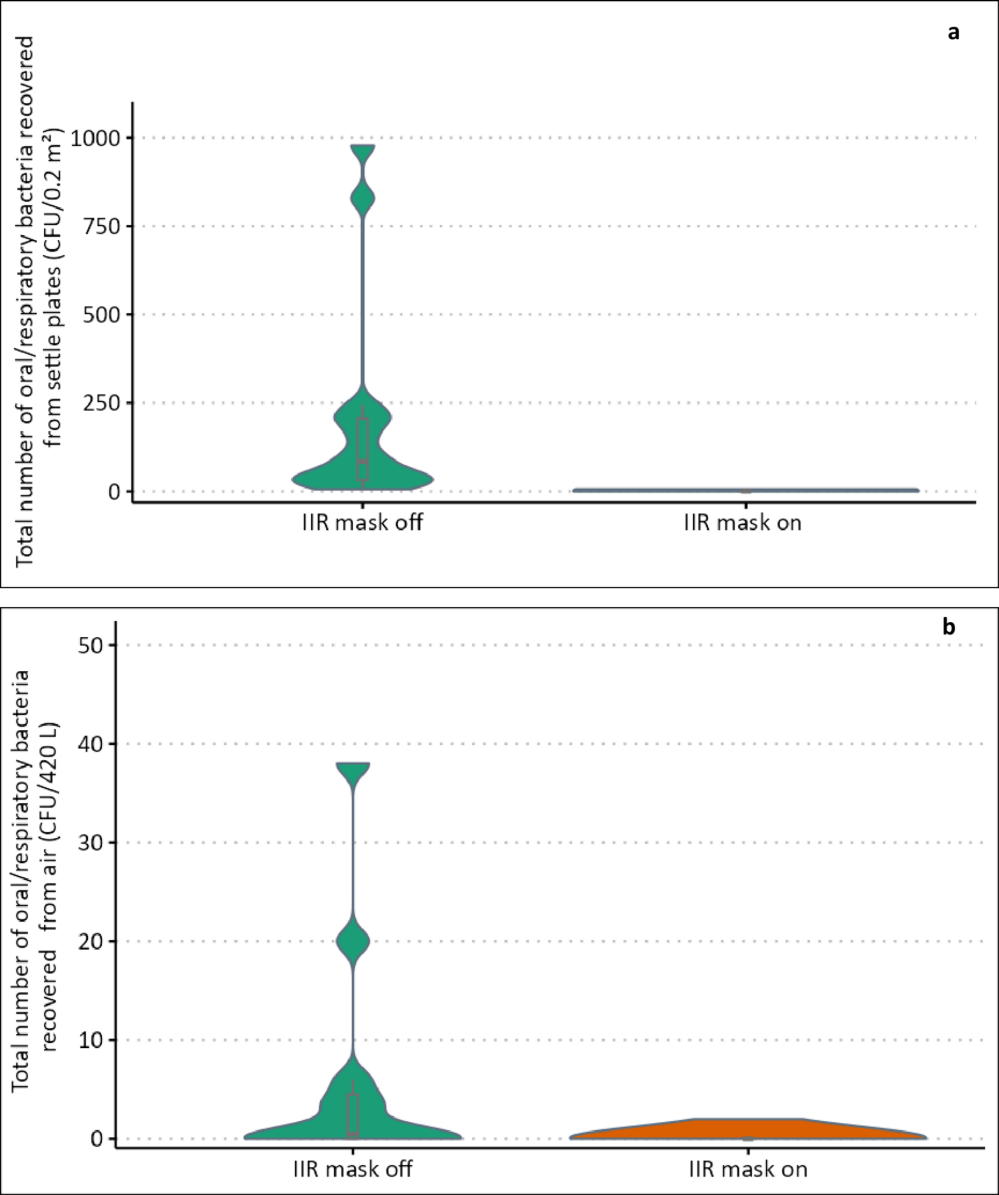
The effectiveness of (IIR) face coverings in reducing the dispersal of small and large respiratory particles (S-RPs and L-RPs). Truncated violin plots with box plot illustrate the total number of URT bacteria deposited on surfaces (L-RPs) within 1 m from source (**a**) and captured by the air sampler (S-RPs) (**b**) positioned 1.2 m from source and operating at 17 L/min when volunteers (*n* = 15) were asked to perform a series of respiratory activities when wearing and when not wearing a (IIR) medical face mask.

Carrying out the same series of respiratory activities whilst wearing a disposable (IIR) medical face mask reduced the median number of URT bacteria deposited as L-RPs to 1 CFU (range: 0 CFU to 6 CFU; p = < 0.004, Fig. 5a) and reduced the odds of detecting higher numbers of S-RPs (1.2 m from source) by 87% (95% CI: 63%, 96%; Fig. 5b).

**Fig. 6:**
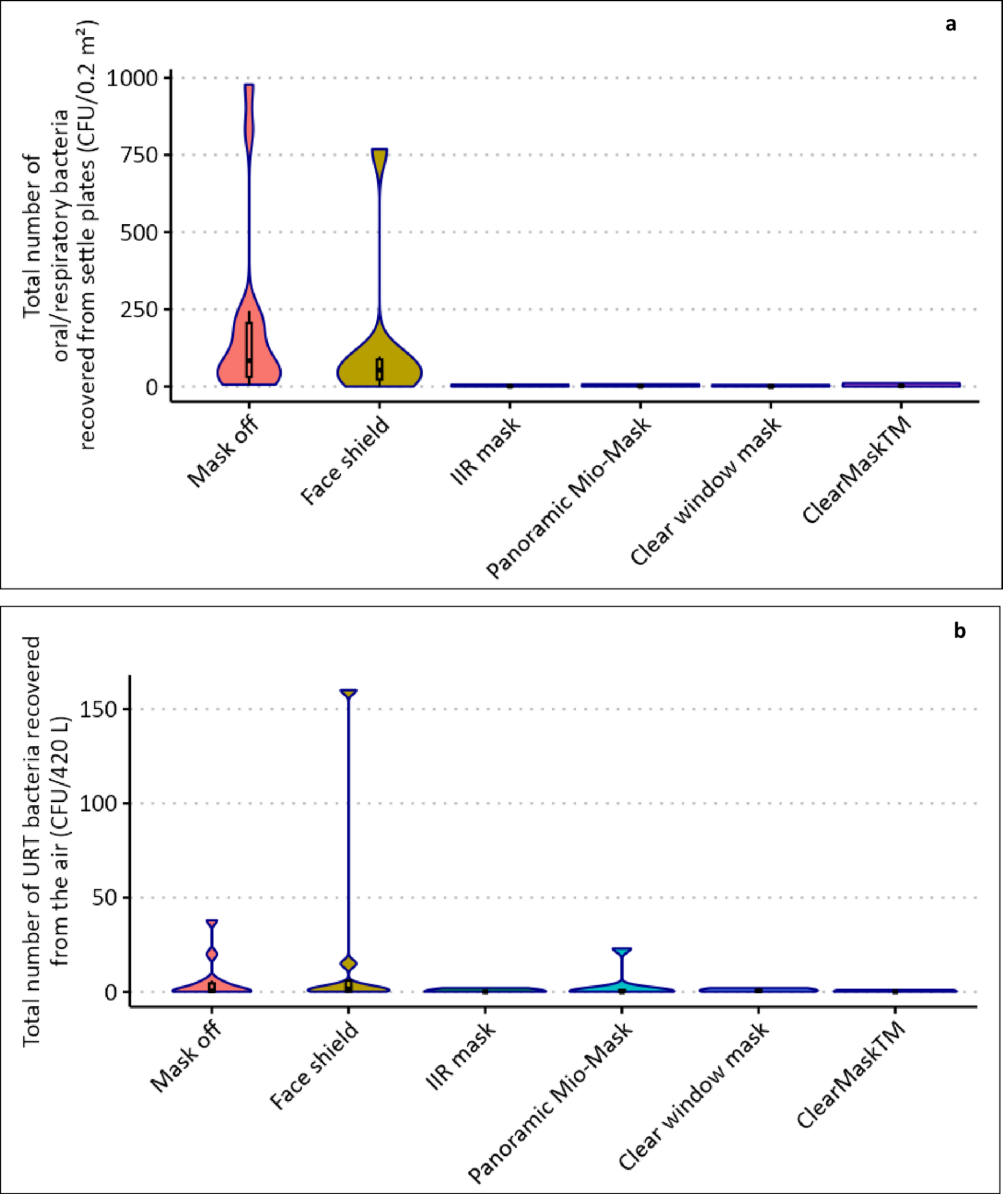
The effectiveness of face coverings in reducing the dispersal of small and large respiratory particles (S-RPs and L-RPs). The total number of URT bacteria deposited on surfaces (L-RPs) within 1 m from source (**a**) and captured by the air sampler (S-RPs) (**b**) when volunteers (*n* = 15) were asked to perform a series of respiratory activities when not wearing a face covering, when wearing a disposable (IIR) medical mask or a randomly assigned transparent face covering.

When volunteers wearing a face shield carried out the same series of respiratory activities, the median number of URT bacteria deposited within 1 m from the source was 54 CFU (range: 0–769 CFU). In contrast, when a reusable clear window mask, a ClearMask™ or a Panoramic Mio-Mask (Fig. 2) was worn, the median number of URT bacteria recovered was ≤ 2 CFU (range: 0–11 CFU) suggesting that wearing any of these transparent face coverings (TFCs) would be as effective as a (IIR) medical face mask in preventing dispersal of L-RPs (Fig. 6a).

Wearing a face shield increased the odds of capturing higher numbers of URT bacteria as S-RPs but not significantly (OR 2.62, CI: 0.51, 13.5). In contrast, wearing any of the TFCs significantly reduced the odds of detecting higher numbers of S-RPs. The reusable clear window mask was the most effective, significantly reducing the odds by 99% (CI: 91% − 100%) (Fig. 6b).

#### Inter-individual variability in dispersal of RPs

In cohort 1 (*n* = 16), individuals with elevated (log) concentrations of URT bacteria in their saliva had increased odds of dispersing higher numbers of L-RPs (95% CI: 1.22, 2.03). However, no significant association was observed with S-RPs. Gender had no significant impact on the dispersal of S-RPs, but after adjusting for height, activity, and dB level, the odds of recovering higher numbers of S-RPs increased by 9% for every year increase in age (95% CI: 1.00–1.19.00.19). Older individuals also had significantly higher odds of dispersing higher numbers of L-RPs.

**Fig. 7:**
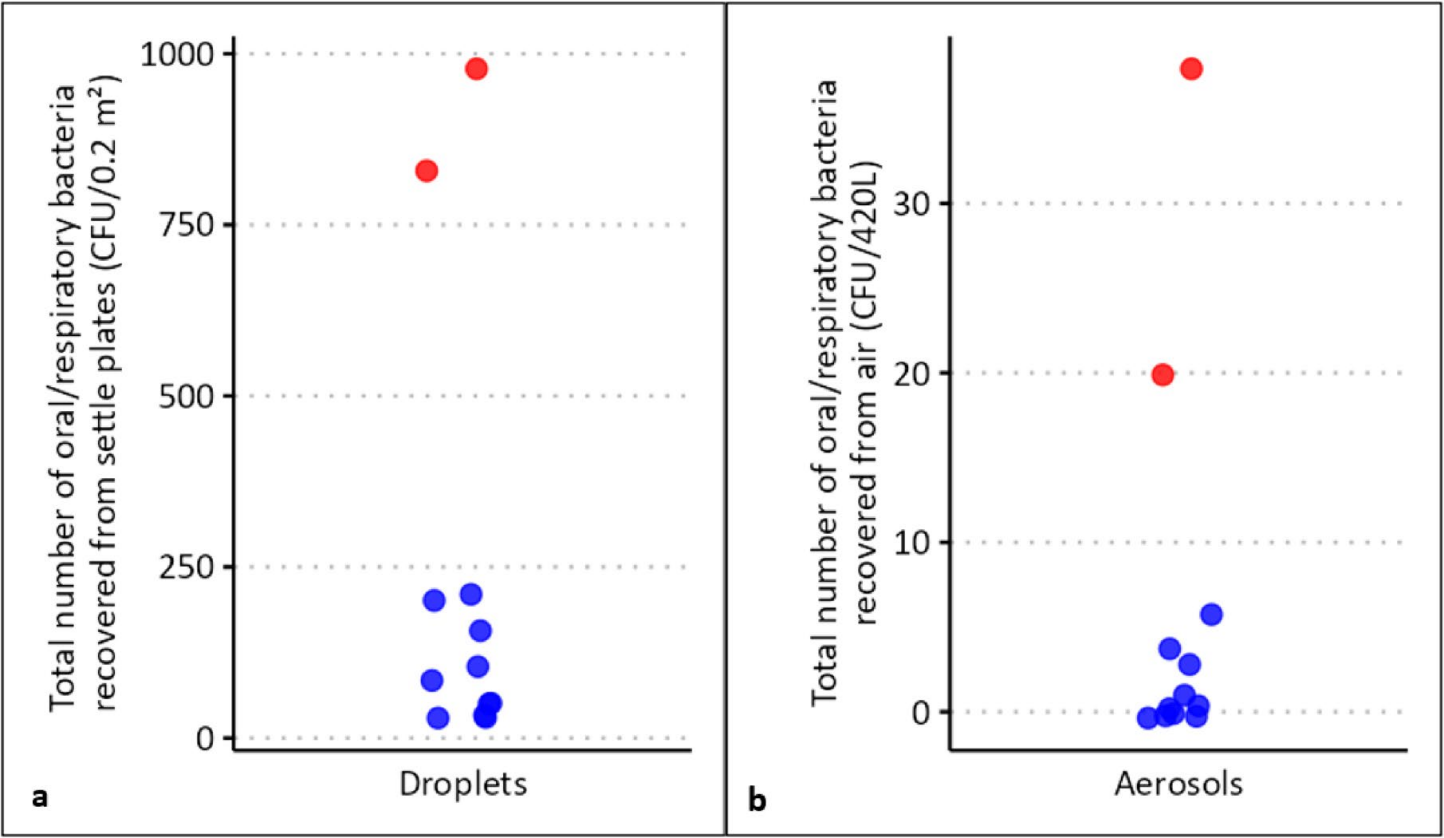
Inter-individual variability in dispersal of respiratory particles (RPs). Two participants (cohort 2) generated 66% and 81% of the URT bacteria deposited on surfaces (L-RPs) (**a**) and captured by the air sampler (S-RPs) (**b**) respectively.

In cohort 2 (*n* = 15), we observed a non-significant trend toward a U-shaped association between the (log) salivary concentration of URT bacteria and dispersal of L-RPs (*p* = 0.08). There was also a significant U-shaped association between age and the odds of detecting higher numbers of S-RPs. In this cohort, the number of L-RPs generated by study participants again differed with gender, with the male participants dispersing seven times more URT bacteria than the females (Supplementary Fig. 2). Two of the male participants generated 66% of all the URT bacteria deposited as L-RPs and 81% of the URT bacteria captured as S-RPs (Fig. 7). When these were removed as outliers or ‘super-emitters’, and by univariant analysis only, the remaining male participants still dispersed significantly more URT bacteria than the females (*p* = 0.04).

## Discussion

Investigating respiratory pathogen transmission presents numerous challenges. Mathematical modelling has been used to better understand transmission patterns of respiratory illness^35,36^ but lacks direct comparability to the complexities of living systems. In vivo models, which often involve animals^37^ or human challenge^38^ are expensive and subject to lengthy ethical approvals.

We propose using upper respiratory tract (URT) bacteria as index organisms for respiratory pathogens. Such an approach overcomes many of the limitations associated with pathogen-based studies and non-microbiological methods of assessing the spread and dispersal of RPs, and provides a practicable, ethical, and scalable alternative for investigating respiratory transmission.

In healthy individuals, the microbial community of the lung is indistinguishable from that of the upper airways^29^ meaning that, in our study, the origin of the bacteria expelled by study participants could not be determined. However, it is likely, due to the type of respiratory activity performed, that the majority of the bacteria detected originated from the upper respiratory tract^39^.

Although viable SARS-CoV-2 is less frequently isolated from saliva compared to nasal or nasopharyngeal samples^40,41^, it has nonetheless been cultured from saliva^42,43^ and other URT secretions^31,32,44^. Active viral replication in minor salivary and parotid glands has also been demonstrated^45^. These findings, although specific to SARS-CoV-2, highlight the potential for saliva and URT secretions to serve as reservoirs for respiratory pathogens and for commensal URT bacteria, which are readily detected in oral fluids, to be used to study the risk of respiratory transmission.

To demonstrate the applicability of this approach, we assessed the impact of increased vocal effort on the generation and dispersal of respiratory particles and compared our findings to those of previous studies^4,12^. Use of URT bacteria confirmed the expected increase in L-RPs and S-RPs and provided a straightforward alternative to more complex methods.

During the COVID-19 pandemic, the mandating of face coverings in public places was a major public health strategy that aimed to mitigate the spread of the virus. Studies using techniques such as aerodynamic particle sizing^12,46^, laser imaging^15^, and mathematical modelling^47–50^ suggested that disposable (IIR) medical face masks would be effective in reducing transmission of SARS-CoV-2. Pathogen-based studies assessing the dispersal of IRPs from human volunteers have yielded similar findings. For example, when wearing a (IIR) medical face mask and when coughing directly onto a Petri dish containing viral media, patients with symptoms of influenza dispersed lower levels of detectable viral RNA in L-RPs than when no face covering was worn^20^. Studies using a Gesundheit device have also demonstrated that face coverings, including IIR medical face masks, can substantially reduce the emission of viral RNA in larger respiratory particles, while being less effective for smaller size fractions^22,23^. Consistent with this, it has been shown that smaller particles are released to a greater extent and can escape from all sides of the mask, with the largest proportion leaking from the top around the nose^46^. Nevertheless, even with such leakage, (IIR) face masks still provide substantial overall source control^46^. Studies measuring viral RNA require participants to be infected with the target virus. Our approach, utilizing commensal bacteria and healthy participants, confirmed the effectiveness of (IIR) medical face masks in significantly reducing the release of L-RPs and S-RPs whilst mitigating the infection risks associated with pathogen-based methods.

Facial expressions are a key component of nonverbal communication - a particularly important aspect of effective communication. During the pandemic, the ubiquity of face masks in healthcare and other settings, created a barrier to effective communication and, inadvertently, an increase in communication stress^51^. While this is of relevance throughout the general population, it is particularly significant for individuals who rely on visual cues, such as those who are hard of hearing. A technical specification for transparent face coverings was originally developed by the NHS Transparent Face Mask Working Group (NHS England and NHS Improvement) in 2021 to provide guidance on performance. At the time, limited data were available on their effectiveness for source control and respiratory particle dispersal, so performance assumptions were made. This specification was withdrawn in March 2023 as it was considered out of date.

A study using a dummy head and breathing simulator demonstrated the functional efficiency of a clear window mask, albeit with reduced source control for smaller particles^52^. Similar studies have highlighted the comparative ineffectiveness of face shields, particularly with the movement of the head^53,54^; a finding also predicted by computational fluid dynamics models^55^.

Using URT bacteria as index organisms, we also demonstrated that when worn by human participants, the three transparent face coverings (clear window mask, the ClearMask™, and the Panoramic Mio-Mask) were as effective in capturing L-RPs and S-RPs as the (IIR) medical face mask. In contrast, face shields, when used as source control, had limited efficacy (Fig. 6), particularly when worn by individuals who dispersed high numbers of URT particles (Supplementary Fig. 3).

Throughout our study, significant inter-individual variability in URT particle dispersal was observed, which aligns with previous findings^12,17,38^. This variability supports the “superspreader” concept, where a small proportion of individuals contribute disproportionately to particle dispersal^56^. While the exact causes remain unclear, our results and those of others suggest that age may play a role, with older individuals potentially dispersing more particles^57–59^. In our study, males dispersed significantly more URT bacteria than females. This gender-related observation is supported by the findings of Yan et al.^21^ and Yang et al.^60^ who also reported increased droplet concentration with age. Other factors that have been linked to ‘super-emitting’ individuals include lung capacity, specifically peak expiratory flow^38^ and obesity^57,58^.

When investigating the dispersal (and transmission) of respiratory pathogens, it is important to acknowledge the potential for high inter-individual variability. The presence (or absence) of ‘super-emitting’ individuals within a study cohort will influence the data obtained and, importantly, its interpretation. To better understand when individuals may expel and disperse more infectious respiratory particles, there is first a need to understand why. More studies are needed to clarify respiratory emission dynamics, and our approach of using URT bacteria as index organisms provides a useful tool to further explore factors that drive individual variation.

In conclusion, we describe a relatively simple, affordable, and accessible method for measuring the dispersal of large- and small RPs. The techniques employed allowed us to confirm that increased vocal effort increases the emission of L-RPs and S-RPs, and that (IIR) medical face masks and transparent face coverings (excluding face shields) can effectively prevent their spread. We identified ‘super emitters’ among those participating in this study and, as in previous studies, observed an association between age and particle dispersal, and showed that face coverings have the greatest impact when worn by high emitters.

However, it is acknowledged that whilst URT bacteria can be considered appropriate index organisms for bacterial pathogens, data may not necessarily translate to respiratory viruses. Infections caused by *Streptococcus pyogenes*, specifically invasive Group A streptococcal (iGAS) disease, can induce severe clinical manifestations and are not infrequent^61^. Our results demonstrate that wearing a (IIR) medical face mask (or transparent face covering) significantly reduces the dispersal of oral streptococci - a potential basis for future recommendations. The respiratory tract harbours a less explored, yet rich virome consisting of high concentrations of non-pathogenic viruses. Among these viruses is Torque teno virus, previously suggested as a potential marker for SARS-CoV-2 infection resolution^62^ and intensive care unit admissions^63^. Current work is investigating the utility of commensal viral markers.

Whilst acknowledging the constraints of our study, including a small cohort size, limited age diversity, and the dimensional constraints of IMADGENN potentially impacting the rate of aerosol decay, we believe its use together with commensal URT bacteria, including streptococci, represents a rapid and efficient system for examining patterns of dispersal (who, why, when) and mitigation strategies for respiratory pathogens. Our current research is expanding upon our findings and further developing and refining this methodology for broader application in the study of respiratory pathogen dispersal.

## Supplementary Information

Below is the link to the electronic supplementary material.

Supplementary Material 1

## Data Availability

The raw data generated for this study are available in Figshare repository – https://doi.org/10.6084/m9.figshare.27159852.v1.
