# Supplementary material for: Assessing the generation and dispersal of respiratory particles using upper respiratory commensal bacteria as index organisms for respiratory pathogens
Source: Sci Rep. 2025 Dec 24;15:44605. doi: 10.1038/s41598-025-28373-z (PMC12738709; doi:10.1038/s41598-025-28373-z)
Supplement: Supplementary file 1 — Supplementary Material 1 [file 41598_2025_28373_MOESM1_ESM.docx]

**Supplementary Material**

**Introduction**
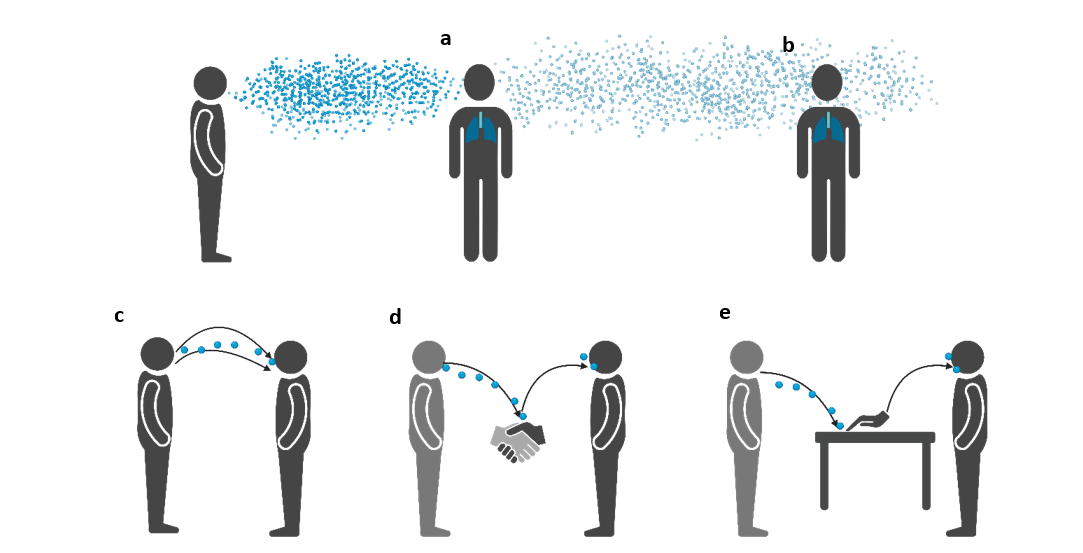


**Supplementary Figure 1: Potential transmission routes of infectious respiratory particles (IRPs).** Short-distance (a) and long-distance (b) airborne transmission both leading to inhalation of IRPs into the respiratory system of a susceptible individual. Semi-ballistic airborne transmission (c) resulting in the deposition of IRPs on the facial mucosal surfaces of the recipient. Direct (d) and indirect (e) contact transmission both followed by transfer of IRPs to the mucosal surfaces of a susceptible individual. Figure created in Biorender.com and adapted from [2]

**Methods**

**Data Analysis**

Modelling strategy

Two of the explanatory variables were gender and facial hair, from which a three-category combined variable (female, male with no facial hair and male with facial hair) was created, as were the logarithm of mean saliva count and age. For cohort 1, oral bacterial counts in both sets of data showed a strong left skewness, with a high proportion of low values, zero in particular. An ordinal variable with three (0, 1-10, >10) and four (0, 1-10, 11-50, >50) categories for the aerosol and droplet data, respectively, was thus created. For cohort 2, the corresponding categories were 0, 1, 2 or more.

In models with at least one continuous fixed effect, the appropriate functional form on the analysis scale was ascertained among the set of linear, quadratic and cubic functions, starting with the last-mentioned and simplifying to that not fitting significantly worse than the next most complex function, while keeping all other (if any) continuous functions as linear or polynomial ascertained if not yet considered or considered, respectively. If any parameter estimates were unstable for a particular functional form, the next simplest function was investigated, regardless of apparent improvement in fit of the more complicated function, as such instability was deemed evidence of model over-fitting and thus unsuitable.

Modelling procedure

Cohort 1

For the droplet data, the initial model consisted of age, the combined variable, height, log saliva, CBA plate position (left, right, centre), distance (10, 20, 30, 40, 50, treated as categorical), activity (speaking, shouting), month (October, November), with the continuous variables age, height, and log saliva. The functional form of the continuous variables was considered in the order just given. Following this, two-way interactions between activity and position, distance and log saliva were considered one at a time, in that order. After this, the mean decibel level variable was entered as continuous, and its appropriate functional form determined using the approach described above. The minimum and maximum decibel level variables were omitted as the mean was derived from them.

For the aerosol data, a series of univariate models consisted of just one fixed effect among the variables listed for the initial droplet model, mean decibel level and three environmental variables. Age and the combined variable were taken forward to the multivariable analysis as potential confounders, as well as those with a p-value of less than 0.2. The procedure described for the droplet data was then followed on a model with these fixed effects without mean decibel level, at the end of which mean decibel level was added and its appropriate functional form ascertained. As the models forming the testing procedure of Williams did not converge, and because the alternative test yielded a highly significant p-value, the multinomial logit model in place of the ordinal logistic regression model failed to produce any estimable standard errors and the fact that the standard errors for the combined variable categories were large and the Wald p-values above 0.05 (albeit only marginally), the combined variable was removed from the ordinal logistic regression model and the simpler ordinal logistic regression model refitted.

Cohort 2

The initial model consisted of each fixed effect as a main effect, with all but one of the continuous variables entering linearly (either age or if it produced a simpler relationship, its logarithm). The fixed effects were (logarithm of) age, logarithm of saliva, CBA plate position, type of face covering (six categories, including no covering), month of test (three categories), relative humidity and the combined variable. The process described in the “Modelling strategy” section was followed for attaining the appropriate functional form of the continuous variables. This was followed by a series of models, each considering one interaction between face covering and, in turn, one of the other factors in the study hypothesis. When considering the aerosol data, the analysis did not include the month of test as there would have been too many parameters in the model relative to number of observations. Those interactions found to be highly significant (p<0.01) were then included in the same model, if possible, and each interaction tested. Each non-significant interaction (at the 1% level) was removed in a stepwise fashion, the conclusion of which yielded the final model, from which p-values were obtained.

In those models where face covering appeared in more than one interaction, a series of models were fitted with just one interaction each, with the other factors that had appeared in interactions being entered as main effects only, in order to obtain the estimates and 95% CIs for the effect of face covering for each of the interaction factors, with the estimates for the remaining variables being those in the final model. This was the case except when the factor was relative humidity, when the effect per unit increase in this variable for each type of face covering was obtained from the final model. An extra step of examining the three-way interaction between face covering, distance and position was inserted after the examination of each two-way interaction separately for the droplet data to determine whether it was significant (at 5% level).

**Results**


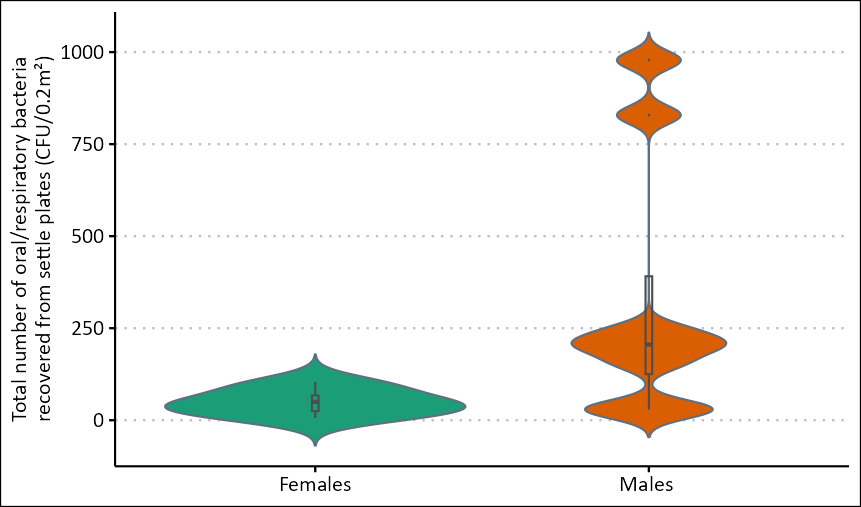
 **Supplementary Figure 2:**  The total number of respiratory bacteria recovered from droplets deposited within 1 m from source when male (n=8) and female (n=7) volunteers were asked to perform a series of respiratory activities. No face covering was worn.

**
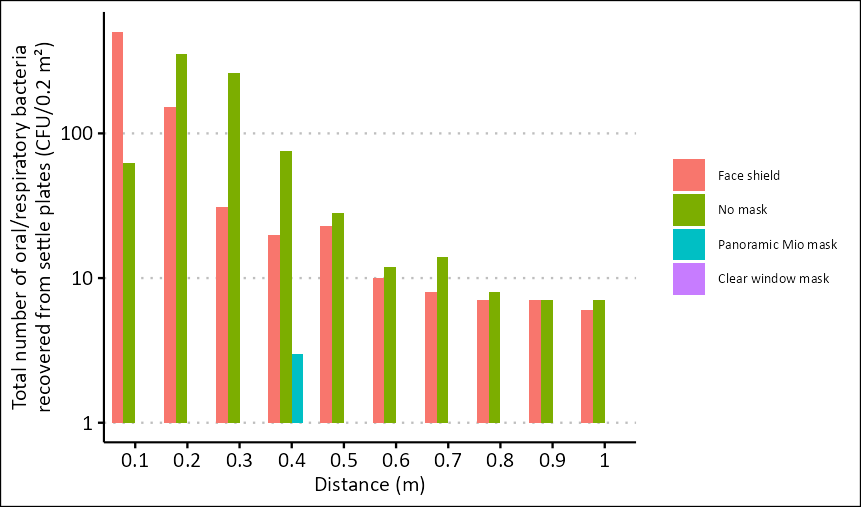
**

**Supplementary Figure 3** The total number of respiratory bacteria recovered from respiratory droplets dispersed by the same healthy volunteer when asked to perform a series of pre-defined respiratory activities when wearing no face covering, a face shield, or a transparent face covering (either a reusable clear window mask or Panoramic Mio-mask).
